# The impact of stringent prescription-only antimicrobial sale regulation (Schedule H1) in India: an interrupted time series analysis, 2008–18

**DOI:** 10.1093/jacamr/dlaa076

**Published:** 2020-10-03

**Authors:** Habib Hasan Farooqui, Sakthivel Selvaraj, Aashna Mehta, Manu Raj Mathur

**Affiliations:** 1 Public Health Foundation of India, Plot No. 47, Sector 44, Institutional Area, Gurugram 122001, India; 2 University of Liverpool, Room B123, Waterhouse Building, Brownlow Street, Liverpool L69 3GL, UK

## Abstract

**Objectives:**

To assess the impact of Schedule H1 regulation notified and implemented in 2014 under the amended rules of the Drugs and Cosmetics Act (DCA), 1940 on the sale of antimicrobials in the private sector in India.

**Methods:**

The dataset was obtained from the Indian pharmaceutical sales database, PharmaTrac. The outcome measure was the sales volume of antimicrobials in standard units (SUs). A quasi-experimental research design—interrupted time series analysis—was used to detect the impact of the intervention.

**Results:**

We observed a substantial rise in antimicrobial consumption during 2008–18 in the private sector in India, both for antimicrobials regulated under Schedule H1 as well as outside the regulation. Key results suggested that post-intervention there was an immediate reduction (level change) in use of Schedule H1 antimicrobials by 10% (*P *=* *0.007), followed by a sustained decline (trend change) in utilization by 9% (*P *>* *0.000) compared with the pre-intervention trend. Segregated analysis on different antimicrobial classes suggests a sharp drop (level changes) and sustained decline (trend changes) in utilization post-intervention compared with the pre-intervention trend. Our findings remained robust on carrying out sensitivity analysis with the oral anti-diabetics market as a control. Post-intervention, the average monthly difference between antimicrobials under Schedule H1 and the control group witnessed an immediate increase of 16.3% (*P *=* *0.10) followed by a sustained reduction of 0.5% (*P *=* *0.13) compared with the pre-intervention scenario.

**Conclusions:**

Though the regulation had a positive impact in terms of reducing sales of antimicrobials notified under the regulation, optimizing the effectiveness of such stand-alone policies will be limited unless accompanied by a broader set of interventions.

## Introduction

Globally, antimicrobial resistance (AMR) currently accounts for about 700 000 deaths annually.^1^^,^^2^ Research suggests that widespread and inappropriate use of antimicrobials is significantly linked to growing AMR worldwide.^3^^,^^4^ This is particularly true for low- and middle-income countries (LMICs) where overall antimicrobial consumption as well as unnecessary antimicrobial use has accelerated.^5^^,^^6^ Global evidence has suggested that antimicrobial consumption rates in LMICs appear to converge towards high-income economies for the period spanning 2000–15.^7^ Furthermore, it has been noted that the consumption of last-resort antimicrobials such as carbapenems and polymyxins has accelerated sharply in these countries in the same period. In India, per capita consumption of antimicrobials has grown from 13.1 DDDs per thousand inhabitants per day (DID) in 2008 to 16.0 DID in 2012, and this increase has been primarily driven by the utilization of newer classes of antimicrobials such as carbapenems, lincosamides, glycopeptides, cephalosporins and penicillins with β-lactamase inhibitors.^8^

Resistance of pathogens to last-resort antimicrobials has been reported in India, using diagnostic laboratory data for the years spanning 2008–14.^9^ The problem of underuse, overuse and misuse of antimicrobials remains an impediment to saving lives in the country. Inappropriate antimicrobial use includes the use of antimicrobials to treat viral conditions, the use of the incorrect class of antimicrobials, the use of incorrect dosage or route of administration, and the lack of proper adherence to treatment. Antimicrobial prescription and consumption rates in India continue to be lower in comparison with European countries^8^ but prescription rates for broad-spectrum β-lactam antimicrobials are significantly higher in India, especially among children. Nearly one-fifth of all antimicrobials prescribed in the country are for upper respiratory infections, which rarely require treatment with an antimicrobial agent. Frequent use of expensive, newer classes of antimicrobials as compared with the older, more affordable ones in the Indian private sector has also been reported.^10^^,^^11^

Inappropriate use of antimicrobials appears to be driven by supply-side factors in the pharmaceutical market. During 2017, it was reported that a single manufacturer produced penicillin G (narrow-spectrum antimicrobial) compared with 135 companies marketing cefixime (broad-spectrum antimicrobial).^12^ Other supply-side factors include rampant prescribing and use of fixed-dose combinations (FDCs) involving two or more antimicrobials. For instance, it is estimated that 68% of FDCs containing antimicrobials on the Indian market have not been approved by the central drug regulator.^13^ Besides, over-the-counter (OTC) availability of antimicrobials at retail pharmacies and self-medication by patients add to inappropriate antimicrobial use. A systematic review of non-prescription use of antimicrobial medicines found that such practices lead to adverse drug reactions.^14^ The same study also reported a decline in antimicrobial use and resistance upon the introduction of regulation. Interventions considered to reduce AMR prevalence include, among others, improving sanitation, public awareness campaigns, promoting new and rapid diagnostics and developing new vaccines.^15^ One of the key interventions is to improve surveillance through monitoring and reporting antimicrobial consumption. A 2018 WHO report called upon member countries to ensure responsible use by way of allowing prescription-only antimicrobials.^16^

In India, the Drugs and Cosmetics Act (DCA), 1940 (and Drugs and Cosmetics Rules, 1945) is the key legislation regulating the import, production, sale, prescription and use of medicines. Schedule H of the DCA governs the sale of prescription-only medicines including antimicrobials. All medicines under Schedule H are prescription only and cannot be purchased over the counter. However, evidence from India suggests that in addition to non-scheduled drugs, antimicrobials on Schedule H were also available over the counter at private pharmacies without a prescription.^17^ In 2013, a new Schedule H1 was introduced under the amended Drugs and Cosmetics Rules to regulate the sale and use of 46 drugs. Schedule H1 was largely intended to regulate newer classes of antimicrobials such as third- and fourth-generation cephalosporins, besides certain habit-forming drugs and anti-TB drugs.^18^ Accordingly, Schedule H1 requires prescribers to record the prescription and patient profile, and this record is expected to be preserved for a period of 3 years. It also directs manufacturers to display labels on the top-left corner of the packet with the symbol Rx, along with a statutory warning with the caution not to use without medical advice and not to be sold without a prescription by a Registered Medical Practitioner. We investigated the impact of the Schedule H1 regulation notified in 2014 under the amended rules of the DCA, 1940 on the sale of antimicrobials in the private sector in India.

## Materials and methods

### Data

The dataset for this analysis was obtained from the Indian pharmaceutical sales database, PharmaTrac, which is a market research company set up as a joint venture between All Indian Origin Chemists and Distributors Ltd (AIOCD Ltd) and Trikaal Mediinfotech Pvt Ltd. The dataset spans a 132 month period from January 2008 to December 2018. PharmaTrac data are collected from a panel of 9000 stockists (about 60% of the total stockists in the country) across 30 different regions of the country and extrapolated to reflect the overall medicine sales in the Indian private-sector retail segment. PharmaTrac datasets are utilized extensively by the Government of India in regulating essential medicine prices.^19^ Medicines are classified and arranged in the dataset based on the anatomical therapeutic chemical (ATC) classification of the European Pharmaceutical Market Research Association (EphMRA). This classification was employed to identify retail sales of antimicrobials, especially Schedule H1 medicines, in the Indian private sector. These belonged to two classes within the anti-infectives segment, i.e. systemic antimicrobials and antimycobacterials. Within the class ‘systemic antimicrobials’, we identified medicines in the subsegments involving cephalosporins, fluoroquinolones, macrolides and similar types, other β-lactams excluding penicillins and cephalosporins, such as monobactams and carbapenems, and other antimicrobials. Within the class ‘antimycobacterials’, two subsegments were identified: anti-TB products and drugs for the treatment of leprosy. This dataset does not capture public-sector utilization of medicines in India, but it does capture the sales of medicines prescribed in the public-sector facilities and bought by patients at retail pharmacies in the private sector. Our analysis therefore focuses exclusively on the impact of the policy on the Indian private-sector retail sales of Schedule H1 antimicrobials. It may also be noted that, in value terms, about 85%–90% of all medicine prescriptions in India occur in the private sector, while the remainder are prescribed, procured and dispensed in government health facilities.^20^

### Intervention under study

The intervention of interest is the notification and subsequent implementation of Schedule H1 of the Drugs and Cosmetics Rules, 1945 (amended 2013). Though Schedule H medicines were already required to be sold with the warning ‘to be sold by retail on the prescription of a Registered Medical Practitioner only’ and labelled with the symbol NRx in red in the top-left corner of the label, Schedule H1 medicines are supposed to be labelled with the symbol Rx in red in the top-left corner of the label along with the warning ‘it is dangerous to take this preparation except in accordance with the medical advice’ and ‘not to be sold by retail without the prescription of a Registered Medical Practitioner’ in a box with a red border. In addition, the dispensing pharmacist is required to maintain a separate register wherein identity of the patient, prescribing doctor’s contact information, name and dispensed quantity of these medicines are recorded. Such a sales register must be retained with the pharmacists for a 3 year period that can be accessed by government drug inspectors during surprise checks.

Thirty-five formulations of antimicrobials were notified under Schedule H1 (see Table S1, available as Supplementary data at *JAC-AMR* Online). Of these, 33 were identified in PharmaTrac data. It must be noted that all the different salt forms as well as FDCs containing these molecules came under the ambit of Schedule H1 and were therefore coded into the dataset as Schedule H1 medicines. Schedule H1 was implemented after 6 months from notification, i.e. 1 March 2014 onwards.

### Outcomes

The primary outcome measure for the study was sales volume of antimicrobials under Schedule H1 regulation in terms of standard units (SUs). SUs are defined as the smallest dose of formulation, which can be one tablet or capsule for oral solids and one vial or ampoule for injectable drugs. We computed the combined sales volumes in SUs of all Schedule H1 antimicrobials for the time period under study and then converted sales volumes into logarithmic form to investigate the change in sales volumes during pre- and post-intervention periods. Antimicrobial sales volume was considered a proxy for antimicrobial consumption for the purpose of this study due to the unavailability of data on actual consumption.

### Research design

We used interrupted time series,^21^ a quasi-experimental research design, to measure the impact of Schedule H1 of the Drugs and Cosmetics Rules, 1945 (amended and notified on 30 August 2013) on antimicrobial utilization in India. A reference market, oral anti-diabetics, which was outside this regulation, was used as a control group to further strengthen our research design.

### Statistical analysis

We performed segmented linear regression analysis to detect the pre-intervention trend, post-intervention level and trend change relative to the pre-intervention level and trend of Schedule H1 antimicrobial utilization. The dependent variable (Yt) appeared as ‘logarithm of sales volume’ of Schedule H1 antimicrobials. ‘Time’ appeared as an independent variable. A least-squares regression line was fitted to the two segments of the continuous variable time and two binary variables were introduced to estimate the immediate level change (variable name: intervention) and trend change (variable name: time after intervention) after the intervention in the logarithm of the sales volume of Schedule H1 antimicrobials (see Equation 1). The Schedule H1 regulation parameter ‘intervention’ is a binary variable taking the value ‘0’ for the 74 month pre-intervention period and the value ‘1’ for the 58 month post-intervention period starting March 2014, whereas ‘time after intervention’ is a continuous variable beginning March 2014. Another dummy (d) was introduced to factor the role of seasonality in consumption. Accordingly, the dummy variable is assigned a value ‘1’ for 3 months (August, September and October) each year. These months were chosen to reflect the monsoon season in India. The choice of the seasonal dummy is consistent with the findings of earlier studies.^8^ The segmented regression analysis helped us statistically determine the change in the intercept (β2) and the slope coefficients (β3) between the pre- and post-intervention periods. α is the baseline intercept and εt is the error term.

(1) Yt=α+β1 time t+β2 intervention t+β3 time after intervention t+d+εt

We also introduced a counterfactual into the model, i.e. the trend in the consumption of Schedule H1 antimicrobials in the post-intervention period had the schedule not been notified. This was done by assuming that the pre-intervention trend would have continued in the post-intervention period had the intervention not been implemented. We checked the model for autocorrelation with the help of the Durbin–Watson statistic and reported autocorrelation and partial autocorrelation estimates and plots of the residuals (see Figures S1, S2 and S3).We detected first-order autocorrelation in our model and therefore altered it to the Prais–Winsten model (Model 2) that makes use of the generalized least-squares method to estimate parameters.

We further refined our model by excluding the adjustment period of 6 months between the date of notification, 30 August 2013, and the date of implementation, 1 March 2014. As part of the sensitivity analysis, we ran our models with a comparison group, i.e. oral anti-diabetics, which was outside the ambit of Schedule H1 regulation, to control for time-varying confounders and other policies that may be impacting intervention and control groups. We ensured that those oral anti-diabetics that came under the ambit of a separate price-regulation policy were excluded before using the segment as a comparison group. This was done to eliminate any confounding effect of price regulation. The analysis was carried out using STATA software version 14.

### Ethics

This study did not entail primary data collection. Secondary pharmaceutical market data were used. We therefore did not require ethics approval for the study.

## Results

**Figure 1.**
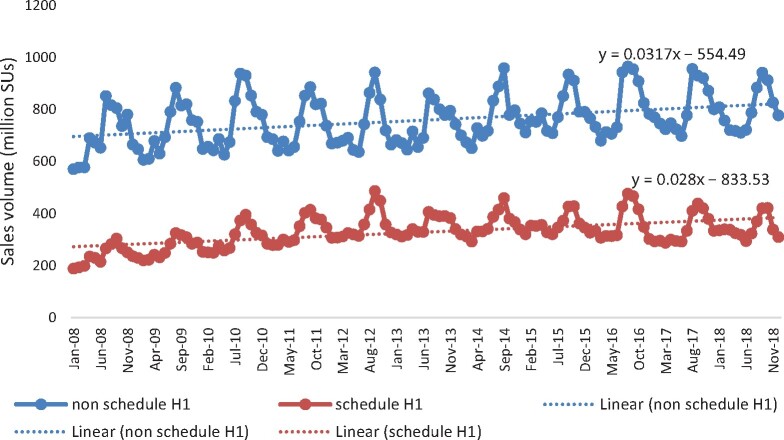
Growth and seasonality in antimicrobial sales.

Our analysis suggests that antimicrobial sales by volume (including antimycobacterials) have been steadily accelerating year on year, with notable peaks reflecting seasonality in consumption, especially in the monsoon season (see Table 1 and Figure 1) in the private retail sector in India during the study period (2008–18). A similar trend was observed in prescription-only (Schedule H1) antimicrobials until 2014, which thereafter remained constant for a couple of years but declined in the later years. As a share, the Schedule H1 antimicrobial sales that were accelerating in the pre-intervention period remained stagnant at about 35% from 2014 onwards.

**Table 1.** Summary statistics, antimicrobial sales in India, 2008–18

	Year										
	2008	2009	2010	2011	2012	2013	2014	2015	2016	2017	2018
Number of producers	611	605	609	511	461	447	585	588	576	586	574
Total antimicrobials	10.07	10.72	11.43	11.66	11.85	11.87	12.04	12.26	12.36	11.68	11.54
Schedule H1 antimicrobials	2.86	3.2	3.64	4	4.27	4.25	4.27	4.31	4.34	4.07	4.13
Share of Schedule H1 antimicrobials (%)	28.42	29.83	31.82	34.31	36.05	35.77	35.48	35.12	35.11	34.89	35.77
Single-ingredient antimicrobials	6.81	7.17	7.55	7.51	7.48	7.35	7.32	7.37	7.57	7.1	7.23
FDC antimicrobials	3.25	3.54	3.88	4.14	4.37	4.52	4.72	4.89	4.8	4.57	4.31
Share of FDC in antimicrobials (%)	32.31	33.06	33.95	35.53	36.91	38.12	39.18	39.87	38.79	39.17	37.35

Volumes are shown in billion SUs.

### Segmented regression model results

Evidence from the segmented regression analysis (Model 1, Table 2) suggests that post-intervention there was an immediate reduction (level changes) in the utilization of Schedule H1 antimicrobials followed by a sustained decline of 9% as compared with the pre-intervention trend. However, we detected first-order autocorrelation in Model 1 (Figures S1, S2 and S3). We corrected for autocorrelation by using the Prais–Winsten model (Model 2). The direction and magnitude of the impact of the intervention remained more or less similar to previous estimates. Results from this model suggest that post-intervention there was an immediate reduction (level change) in the utilization of Schedule H1 antimicrobials by 10% (*P *=* *0.007), followed by a sustained decline (trend change) of 9% (*P *>* *0.000) as compared with the pre-intervention trend.

**Table 2.** Segmented regression model results

Variables	Model 1		Model 2		Model 3	
	coefficient	*P* value (95% CI)	coefficient	*P* value (95% CI)	coefficient	*P* value (95%CI)
Time	0.008	0.000 (0.007–0.01)	0.008	0.000 (0.006–0.010)	0.019	0.000 (0.016–0.022)
Intervention (level change)	−0.08	0.007 (−0.138 to −0.022)	−0.1	0.054 (−0.203 to 0.002)	0.163	0.101 (−0.033 to 0.360)
Time after intervention (trend change)	−0.009	0.00 (−0.010 to −0.007)	−0.009	0.00 (−0.012 to −0.006)	−0.005	0.126 (−0.010 to 0.001)
Seasonal dummy	0.246	0.000 (0.213–0.280)	0.155	0.000 (0.118–0.192)	NA	NA
Constant	19.18	0.000 (19.144–19.224)	19.19	0.000 (19.113–19.268)	18.09	0.000 (17.967–18.220)
Number of observations	126		126		126	
pre-intervention	68		68		68	
post-intervention	58		58		58	
R^2^	0.8238		0.9863		0.8747	

NA, not applicable.

**Figure 2.**
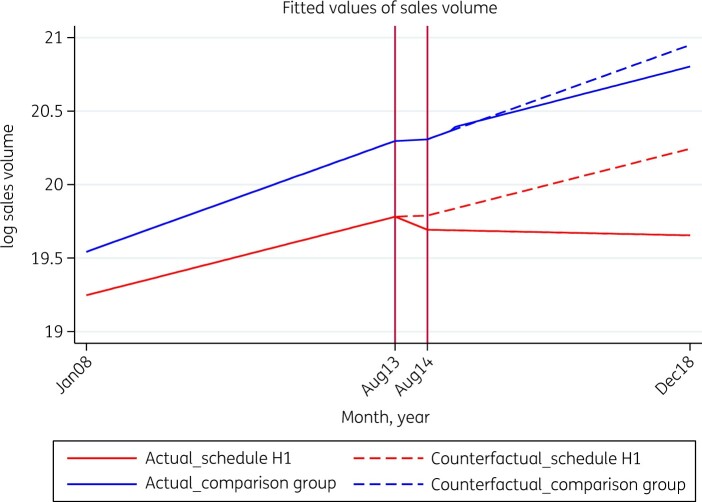
Actual and counterfactual medicine consumption for regulated antimicrobials and control drugs. The comparison (control) group is oral anti-diabetics in the Indian pharmaceutical market.

As part of sensitivity analysis, we carried out another segmented regression analysis (Model 3) with a control group—medicines belonging to the oral anti-diabetics segment—which was outside of this regulation. Table 2 and Figure 2 (Model 3) demonstrate that in the pre-intervention period the average monthly trend in the difference between antimicrobials under Schedule H1 and the control group was 1.9% (*P *=* *0.000). However, post-intervention the average monthly difference between antimicrobials under Schedule H1 and the control group witnessed an immediate increase (level change) followed by a sustained reduction (trend change) as compared with the pre-intervention scenario, but both changes were statistically insignificant.

Figure 2 presents a plot of fitted values of the two groups of medicines—Schedule H1 antimicrobials (treatment group) and oral anti-diabetics (control group)—in the actual (solid lines) and counterfactual (dotted lines) scenarios. The counterfactual represents the scenario had the intervention (Schedule H1) not been implemented. The vertical lines in the figure represent the implementation period (6 month period between the notification of Schedule H1 and its implementation). The figure shows that in the absence of regulation (the counterfactual scenario), Schedule H1 antimicrobial consumption would have witnessed a rising secular trend (dotted red lines). However, as a result of the interventions (the actual scenario), regulated antimicrobial utilization recorded a steep decline (solid red lines). The comparison group too witnessed a decline in utilization in the post-intervention period (solid blue line) in comparison with the counterfactual scenario (dotted blue line). However, the decline was far more pronounced in the treatment group (Schedule H1 antimicrobials) as compared with the control group (oral anti-diabetics).

We further conducted formulation level and subtherapeutic classwise analysis of level and trend change post-intervention. Table 3 highlights those results. Schedule H1 formulations belonging to the cephalosporins, fluoroquinolones and anti-TB classes witnessed a sharp drop and sustained decline in utilization post-intervention. The only exception was Schedule H1 formulations in the class of ‘other β-lactam antimicrobials’, which saw a sharp increase, followed by a small but sustained rise in utilization. The antimicrobials outside Schedule H1 witnessed a sharp rise in use immediately after the intervention. The increase, however, was insignificant. For instance, broad-spectrum penicillin formulations, as well as tetracyclines and combinations that were outside Schedule H1, witnessed a sudden rise followed by a sustained drop in utilization in the post-intervention period in comparison with the pre-intervention period, whereas medium- and narrow-spectrum penicillins, as well as aminoglycosides that were also outside Schedule H1, reported a sudden and sustained drop in utilization in the post-intervention period.

**Table 3.** Segmented regression models under subtherapeutic categories

Subtherapeutic categories	Time (*P* value)	Intervention (*P* value)	Time after intervention (*P* value)	Constant (*P* value)	Goodness of fit (R^2^)	No. of medicines
Classes with Schedule H1 formulations						
cephalosporins	0.012 (0.000)	−0.094 (0.199)	−0.012 (0.000)	18.538 (0.000)	0.989	47
fluoroquinolones	0.002 (0.093)	−0.123 (0.055)	−0.005 (0.007)	17.256 (0.000)	0.991	9
other β-lactam antimicrobials	0.014 (0.000)	0.280 (0.005)	0.002 (0.470)	13.135 (0.000)	0.988	6
anti-TB	0.002 (0.001)	−0.120 (0.003)	−0.006 (0.000)	18.110 (0.000)	0.996	17
total Schedule H1 antimicrobials	0.008 (0.000)	−0.08 (0.0054)	−0.009 (0.000)	19.184 (0.000)	0.986	83
Classes without Schedule H1 formulations						
broad-spectrum penicillins	0.003 (0.002)	0.021 (0.703)	−0.002 (0.296)	18.848 (0.000)	0.994	31
medium- and narrow-spectrum penicillins	0.006 (0.119)	−0.028 (0.907)	−0.007 (0.253)	16.742 (0.000)	0.947	7
aminoglycosides	0.003 (0.013)	−0.024 (0.759)	−0.023 (0.285)	16.230 (0.000)	0.991	8
tetracycline and combinations	0.004 (0.002)	0.045 (0.500)	−0.011 (0.000)	17.964 (0.000)	0.981	12
total antimicrobials outside Schedule H1	0.001 (0.089)	0.009 (0.843)	−0.000 (0.898)	20.318 (0.000)	0.992	136

Schedule H1 cephalosporins include cefixime, cefdinir, cefpodoxime, ceftriaxone, ceftazidime, cefoperazone, cefepime, ceftizoxime, cefpirome, cefetamet, cefotaxime, cefditoren, ceftibuten and their combinations. Schedule H1 fluoroquinolones include balofloxacin, gemifloxacin, levofloxacin, moxifloxacin, prulifloxacin, sparfloxacin and their combinations. Schedule H1 ‘other β-lactam antibacterials’ include ertapenem, faropenem, imipenem, meropenem and their combinations. Schedule H1 anti-TB drugs include capreomycin, cycloserine, ethambutol, ethionamide, isoniazid, pyrazinamide, rifabutin, rifampicin and their combinations. ‘Total antimicrobials outside Schedule H1’ includes all antimicrobials belonging to classes not containing Schedule H1 antimicrobials and also antimicrobials left out of Schedule H1 in classes that include some Schedule H1 antimicrobials such as cephalosporins, fluoroquinolones etc.

## Discussion

This piece of research is, to our knowledge, the first attempt to systematically report the impact of Schedule H1, a stringent version of Schedule H of Drugs and Cosmetics Rules, 1945 (amended 2013), which governs the sales and dispensing of prescription-only medicines in India. Using an interrupted time series regression model, we report the impact of Schedule H1 regulation on utilization of notified antimicrobials in the Indian private sector from 2008 to 2018. We observed that nearly 600 manufacturers were marketing antimicrobials in India, reflecting a competitive market. However, FDCs involving one or more antimicrobials accounted for over one-third of antimicrobials sold in the market.

During our study period, a substantial rise in antimicrobial consumption was observed in the private sector in India, both for Schedule H1 and non-Schedule H1 antimicrobials. In the monsoon season, antimicrobial sales volume in the country peaked on account of various bacterial and viral infections. Earlier studies in India have shown that although overall utilization of antimicrobials was relatively lower *vis-à-vis* European standards when measured as DID, they demonstrated wider usage of third- and fourth-generation antimicrobials, suggesting inappropriate use.^8^

We observed a steep decline in the sales volume of Schedule H1 antimicrobials in the 6 month period between the notification of Schedule H1 and its implementation, which suggests that the suppliers must have been withdrawing the stocks from the market to re-label the medicine packs to comply with the government’s directive. This period was excluded from the model to remove its confounding effect on our results. We found a significant decline in both level and trend of antimicrobial use after Schedule H1 restrictions were imposed in 2014. A negative direction of post-intervention level and trend changes as compared with pre-intervention level and trend was observed among cephalosporins, fluoroquinolones and anti-TB drugs, suggesting a sharp drop and sustained decline in sales volume post-intervention. The segment ‘other β-lactam antimicrobials’, which includes carbapenems and monobactams, witnessed a sharp immediate increase in sales after the intervention compared with the pre-intervention period followed by a small sustained increase in the trend. The sharp but insignificant increase observed for non-Schedule H1 antimicrobials could possibly indicate a small substitution effect of retail sales from restricted to non-restricted antimicrobials.

Similar laws restricting OTC sales of antimicrobials in 2010 in Brazil and Mexico were observed to have resulted in a decline in the overall usage pattern. Specifically, penicillin, sulphonamide and macrolide usage levels indicated in terms of DDD declined in Brazil after the intervention, while in Mexico the consumption of penicillin and sulphonamides was found to have declined significantly.^22^ Another study from these countries suggested that restriction of OTC sales of antimicrobials resulted in unintended consequences by increasing the use of symptomatic drugs including those for colds and coughs and non-steroidal anti-inflammatory drugs (NSAIDs).^23^ A Swedish study revealed a decline in antimicrobial use among pre-school children, as they were less commonly prescribed, but reported an associated increase in paediatric paracetamol sales, suggesting that symptomatic self-treatment had increased.^24^

A key limitation of this study is the inclusion of only private-sector sales data for antimicrobial products. However, it may be noted that in government health facilities such restrictions are superfluous because both procurement and prescriptions are governed by the Essential Medicines List. Moreover, as per the Indian constitution, health service delivery in the public sector is under state government and there are about 35 states in India. No single and uniform database exists or is available for public scrutiny. Furthermore, in value terms about 85%–90% of all medicine prescriptions in India occur in the private sector, while the remainder are prescribed, procured and dispensed in government health facilities.^20^ Thus, the role of an OTC sales ban of antimicrobials is largely relevant to the private sector.

It is also recognized that regulations prohibiting antimicrobial OTC sales may also lead to unintended consequences by means of a substitution effect. Antimicrobials are often prescribed without proper diagnosis, especially in LMICs. For instance, antimicrobials are commonly prescribed for infections such as upper respiratory tract infections that are viral and self-limiting in nature and do not require them. Patients also tend to self-medicate for conditions such as common cold and cough, for symptomatic relief. Although such substitution is potentially expected, the regulation under study is aimed at restricting the use of third- and fourth-generation antimicrobials. It is equally plausible that the use of non-restrictive first- and second-generation antimicrobials would have increased due to this regulation.

### Conclusions

Policy instruments are often deployed to influence the demand and supply of health products and services, through direct or indirect controls. The primary objective of Schedule H1 regulation was to restrict the use of antimicrobials, especially the broad-spectrum antimicrobials, using cautionary labels and symbols on the packages to increase awareness among patients, dispensers and prescribers and encourage them to act with caution. However, the effectiveness of such stand-alone policies could be limited unless accompanied by other interventions. Effective regulation at every stage including import, production, distribution, procurement, prescription, dispensing and use is required. For instance, market authorization of an antimicrobial product must be by a single government agency. This is critical given the fact that around 40% of all antimicrobial products sold in India are FDCs. Another potential solution lies in transforming the current landscape of the health sector towards universal health coverage instead of being dominated by a system of private provisioning and financing. As long as the health sector is financed by households directly, perverse incentives will remain among producers, prescribers and dispensers.

## Funding

This study was carried out as part of our routine research work.

## Transparency declarations

None to declare.

##  

Table S1 and Figures S1 to S3 are available as Supplementary data at *JAC-AMR* Online.

## Supplementary Material

dlaa076_Supplementary_Data

## References

[1] UN, Interagency Coordination Group on Antimicrobial Resistance. No Time to Wait: Securing the Future from Drug-Resistant Infections, Report to the Secretary-General of the United Nations. https://www.who.int/antimicrobial-resistance/interagency-coordination-group/final-report/en/.

[2] The Review on Antimicrobial Resistance. 2016. Tackling Drug-Resistant Infections Globally: Final Report and Recommendations. https://amr-review.org/sites/default/files/160518_Final%20paper_with%20cover.pdf.

[3] Goossens H Ferech M Vander Stichele R et al Outpatient antibiotic use in Europe and association with resistance: a cross-national database study. Lancet 2005; 365: 579–87. 15708101 10.1016/S0140-6736(05)17907-0

[4] Costelloe C Metcalfe C Lovering A et al Effect of antibiotic prescribing in primary care on antimicrobial resistance in individual patients: systematic review and meta-analysis. BMJ 2010; 340: c2096. 20483949 10.1136/bmj.c2096

[5] Van Boeckel TP Gandra S Ashok A et al Global antibiotic consumption 2000 to 2010: an analysis of national pharmaceutical sales data. Lancet Infect Dis 2014; 14: 742–50. 25022435 10.1016/S1473-3099(14)70780-7

[6] Mendelson M Rottingen JA Gopinathan U et al Maximising access to achieve appropriate human antimicrobial use in low-income and middle-income countries. Lancet 2016; 387: 188–98. 26603919 10.1016/S0140-6736(15)00547-4

[7] Klein EY Van Boeckel TP Martinez EM et al Global increase and geographic convergence in antibiotic consumption between 2000 and 2015. Proc Natl Acad Sci USA 2018; 115: E3463–70. 29581252 10.1073/pnas.1717295115 PMC5899442

[8] Farooqui HH Selvaraj S Mehta A et al Community level antibiotic utilization in India and its comparison vis-a-vis European countries: evidence from pharmaceutical sales data. PLoS One 2018; 13: e0204805. 30332450 10.1371/journal.pone.0204805 PMC6192587

[9] Gandra S, Nestor M Klein EY et al Trends in antibiotic resistance among major bacterial pathogens isolated from blood cultures tested at a large private laboratory network in India. Int J Infect Dis 2016; 50: 75–82. 27522002 10.1016/j.ijid.2016.08.002 PMC5063511

[10] Kotwani A Chaudhury RR Holloway K. Antibiotic-prescribing practices of primary care prescribers for acute diarrhea in New Delhi, India. Value Health 2012; 15: S116–9. 22265057 10.1016/j.jval.2011.11.008

[11] Chandy SJ Thomas K Mathai E et al Patterns of antibiotic use in the community and challenges of antibiotic surveillance in a lower-middle-income country setting: a repeated cross-sectional study in Vellore, South India. J Antimicrob Chemother 2013; 68: 229–36. 22945913 10.1093/jac/dks355

[12] CIMS. *Current Index of Medical Specialty (CIMS India), April–June 2017*. CIMS India, 2017.

[13] McGettigan P Roderick P Mahajan R et al Use of fixed dose combination (FDC) drugs in India: central regulatory approval and sales of FDCs containing non-steroidal anti-inflammatory drugs (NSAIDs), metformin, or psychotropic drugs. PLoS Med 2015; 12: e1001826. 25965416 10.1371/journal.pmed.1001826 PMC4428752

[14] Morgan DJ Okeke IN Laxminarayan R et al Non-prescription antimicrobial use worldwide: a systematic review. Lancet Infect Dis 2011; 11: 692–701. 21659004 10.1016/S1473-3099(11)70054-8 PMC3543997

[15] WHO. Global Action Plan on Antimicrobial Resistance. https://apps.who.int/iris/bitstream/handle/10665/193736/9789241509763_eng.pdf?sequence=1.

[16] WHO. WHO Report on Surveillance of Antibiotic Consumption: 2016-2018 Early Implementation. https://www.who.int/medicines/areas/rational_use/who-amr-amc-report-20181109.pdf?ua=1.

[17] Laxminarayan R Chaudhury RR. Antibiotic resistance in India: drivers and opportunities for action. PLoS Med 2016; 13: e1001974. 26934098 10.1371/journal.pmed.1001974 PMC4775002

[18] Government of India. The Drugs and Cosmetics Act, 1940 and The Drugs and Cosmetics Rules, 1945. https://cdsco.gov.in/opencms/export/sites/CDSCO_WEB/Pdf-documents/acts_rules/2016DrugsandCosmeticsAct1940Rules1945.pdf.

[19] Government of India. Annual Report 2019-20, Department of Pharmaceuticals, Ministry of Chemicals and Fertilizers. https://pharmaceuticals.gov.in/sites/default/files/Annual%20Report%202019-20.pdf.

[20] National Health Systems Resource Centre. National Health Accounts: Estimates for India. Financial Year 2015-16. https://main.mohfw.gov.in/sites/default/files/NHA_Estimates_Report_2015-16_0.pdf.

[21] Wagner AK Soumerai SB Zhang F et al Segmented regression analysis of interrupted time series studies in medication use research. J Clin Pharm Ther 2002; 27: 299–309. 12174032 10.1046/j.1365-2710.2002.00430.x

[22] Santa-Ana-Tellez Y Mantel-Teeuwisse AK Dreser A et al Impact of over-the-counter restrictions on antibiotic consumption in Brazil and Mexico. PLoS One 2013; 8: e75550. 24146761 10.1371/journal.pone.0075550 PMC3797702

[23] Santa-Ana-Tellez Y Mantel-Teeuwisse AK Leufkens HG et al Effects of over-the-counter sales restriction of antibiotics on substitution with medicines for symptoms relief of cold in Mexico and Brazil: time series analysis. Health Policy Plan 2016; 31: 1291–6. 27229872 10.1093/heapol/czw066

[24] Hogberg L Oke T Geli P et al Reduction in outpatient antibiotic sales for pre-school children: interrupted time series analysis of weekly antibiotic sales data in Sweden 1992-2002. J Antimicrob Chemother 2005; 56: 208–15. 15897223 10.1093/jac/dki147

