# Supplementary material for: The impact of stringent prescription-only antimicrobial sale regulation (Schedule H1) in India: an interrupted time series analysis, 2008–18
Source: JAC Antimicrob Resist. 2020 Oct 3;2(3):dlaa076. doi: 10.1093/jacamr/dlaa076 (PMC8210317; doi:10.1093/jacamr/dlaa076)
Supplement: dlaa076_Supplementary_Data [file dlaa076_supplementary_data.docx]

**Supplementary data**

**Table S1:** List of Schedule H1 antimicrobials

1. Balofloxacin
2. Capreomycin
3. Cefdinir
4. Cefditoren
5. Cefepime
6. Cefetamet
7. Cefixime
8. Cetoperazone
9. Cefotaxime
10. Cefpirome
11. Cefpodoxime
12. Ceftazidime
13. Ceftibuten
14. Ceftizoxime
15. Ceftriaxone
16. Clofazimine
17. Cycloserine
18. Doripenem
19. Etrapenem
20. Ethambutol Hydrochloride
21. Ethionamide
22. Feropenem
23. Gemifloxacin
24. Imipenem
25. Isoniazid
26. Levofloxacin
27. Meropenem
28. Moxifloxacin
29. Prulifloxacin
30. Pyrazinamide
31. Rifabutin
32. Rifampicin
33. Sodium Para-aminosalicylate*
34. Sparfloxacin
35. Thioacetazone*

*Not available in PharmaTrac data

**Figure S1: Plot of residuals**

**Figure S2: Plot of complete autocorrelation of residuals**

**Figure S3: Plot of partial autocorrelation of residuals**

**Durbin-Watson statistic = 1.53**

**Transformed Durbin-Watson statistic (after Prais-Winsten model) = 2.02**
